# Model Communities Hint at Promiscuous Metabolic Linkages between Ubiquitous Free-Living Freshwater Bacteria

**DOI:** 10.1128/mSphere.00202-18

**Published:** 2018-05-30

**Authors:** Sarahi L. Garcia, Moritz Buck, Joshua J. Hamilton, Christian Wurzbacher, Hans-Peter Grossart, Katherine D. McMahon, Alexander Eiler

**Affiliations:** a Department of Ecology and Genetics, Limnology and Science for Life Laboratory, Uppsala University, Uppsala, Sweden; b Department of Bacteriology, University of Wisconsin—Madison, Madison, Wisconsin, USA; c Department of Biological and Environmental Sciences, University of Gothenburg, Göteborg, Germany; d Institute for Biochemistry and Biology, Potsdam University, Potsdam, Germany; e Department of Experimental Limnology, Leibniz-Institute for Freshwater Ecology and Inland Fisheries, Stechlin, Germany; f Department of Civil and Environmental Engineering, University of Wisconsin—Madison, Madison, Wisconsin, USA; DOE Joint Genome Institute

**Keywords:** community, interactions, metagenomics, microbial ecology, mixed cultures, promiscuous

## Abstract

Genome streamlining is frequently observed in free-living aquatic microorganisms and results in physiological dependencies between microorganisms. However, we know little about the specificity of these microbial associations. In order to examine the specificity and extent of these associations, we established mixed cultures from three different freshwater environments and analyzed the cooccurrence of organisms using a metagenomic time series. Free-living microorganisms with streamlined genomes lacking multiple biosynthetic pathways showed no clear recurring pattern in their interaction partners. Free-living freshwater bacteria form promiscuous cooperative associations. This notion contrasts with the well-documented high specificities of interaction partners in host-associated bacteria. Considering all data together, we suggest that highly abundant free-living bacterial lineages are functionally versatile in their interactions despite their distinct streamlining tendencies at the single-cell level. This metabolic versatility facilitates interactions with a variable set of community members.

## Abstract (precis)

Genome streamlining is frequently observed in free-living aquatic microorganisms and results in physiological dependencies between microorganisms. However, we know little about the specificity of these microbial associations.

## OPINION/HYPOTHESIS

Microorganisms can interact in many different ways, and their relationships range from facultative to obligate dependencies (1). Endosymbionts are at one end of the dependency spectrum, and a defined host-endosymbiont specificity is established in most cases. At the other end of the spectrum, free-living bacteria are widely considered to be autonomous. However, this paradigm is shifting with frequent reports of reduced microbial genomes in the environment resulting in unique and singular auxotrophies (2).

In natural aquatic environments where nutrients generally occur in low concentrations, microbes produce many compounds that are costly but promote survival and reproduction not only for themselves but also for other cells in the community (3). In fact, auxotrophy for amino acids and vitamins, among other things, has recently been reported and discussed for numerous free-living bacteria (4, 5). Thus, individual bacteria in a community likely function as nodes in a network of interacting cells that reciprocally exchange nutrients and biochemical functions rather than act as physiologically autonomous units (6). Therefore, it would not be surprising to find that most natural aquatic systems allow for frequent, variable, and complex metabolic interactions, for example, via continuous mixing of the environment and molecular-scale diffusion facilitating distribution of public goods. Hence, abundant streamlined environmental bacteria are tightly linked and dependent on other microorganisms in the community (7). The sheer number of bacteria and their genetic variability in a population (8, 9) potentially allow for high metabolic flexibility at the population level, with an endless number of metabolic interactions possible among the numerous members of free-living bacterial communities. However, the character of these associations still remains largely unknown.

To investigate the specificity of dependencies between free-living freshwater microorganisms, we focused on a group of cosmopolitan, yet streamlined, freshwater organisms from the actinobacterial lineage known as acI. Actinobacteria of the acI lineage are the most abundant microbes in freshwater systems (10), but there are so far no alive pure cultures of these organisms. However, 20 transient cultures of these bacteria have produced full genomes with potential auxotrophy for various vitamins, amino acids, and reduced sulfur sources (4, 11). These transient cultures did not survive many transfers and/or were not maintainable as monocultures. Therefore, we turned to dilution mixed cultures (methods are found in the supplemental material). These cultures can serve as model communities, as they are a small subsample of the complex natural community (12). We started cultures with about 12 initial cells, and about 400 such mixed cultures were established and screened for acI organisms.

## MIXED CULTURES AS MODEL COMMUNITIES AND THEIR GENOMIC RECONSTRUCTIONS

Cultures were obtained by diluting lake samples with triple-filtered sample water incubated in 96-well liquid culture plates (four in total). Six cultures containing acI organisms were successfully propagated with densities comparable to those observed in the environment (i.e., 10^6^ cells ⋅ ml^−1^) for subsequent transfers and growth in larger volumes. This resulted in 6 dilution-to-extinction mixed cultures: FNEF8, FNEB6, FNEB7, FNED7, FSWF8, and TBE6 (FNE refers to the northeast basin of Lake Grosse Fuchskuhle, FSW to the southeast basin of Lake Grosse Fuchskuhle, and TB to Trout Bog Lake). A detailed characterization of the FNEF8 culture was published previously (13, 14). The fact that mixed cultures containing acI organisms do indeed survive tests and propagations (14) serves as strong support that the free-living acI microorganisms are highly dependent on interactions with other microorganisms.

DNA was extracted from 4 liters of culture, and reads obtained by shotgun sequencing were assembled. Contigs of >1,000 bp were considered for further analysis. In total, 77 metagenome assembled genomes (MAGs) were obtained from the assemblies. Of these, 31 MAGs each recruited more than 1% of the reads in the culture’s metagenome (see Table 1 in Data Set S1 in the supplemental material). The other 46 MAGs recruited less than 3% of all reads combined. Altogether, populations represented by these top 31 MAGs were assumed to be the dominant model community members in each of the cultures from which they were assembled (Table 1). All six cultures yielded at least one MAG of the cosmopolitan freshwater actinobacterial lineage acI (recruiting between 15% and 63% of all reads), four cultures yielded a MAG of the freshwater *Actinobacteria* lineage acIII (between 4% and 9% of all reads), two cultures featured MAGs affiliated with *Bacteroidetes* (3% and 7% of all reads) that were divergent from any described genus, two cultures yielded *Polynucleobacter* MAGs (2% and 56% of all reads), one culture yielded a *Spirochaetes* MAG, *Leptospira* (9% of all reads), one culture yielded a *Spirochaetes* organism divergent from any described genus (22% of all reads), one culture yielded an alphaproteobacterium MAG, *Bradyrhizobium* (2% of all reads), and one culture yielded an *Acidimicrobiales* MAG, *Acidimicrobium* (48% of all reads). Two cultures also yielded yeast MAGs (1 and 4% of all reads). Both yeasts in the cultures (*Rhodotorula* and *Aureobasidium*) are ubiquitous in nature and also occur normally in surface waters (15). It is very interesting that, in our cultures, we observed potential cross-domain linkages to these two ubiquitous yeasts.

**FIG 1:**
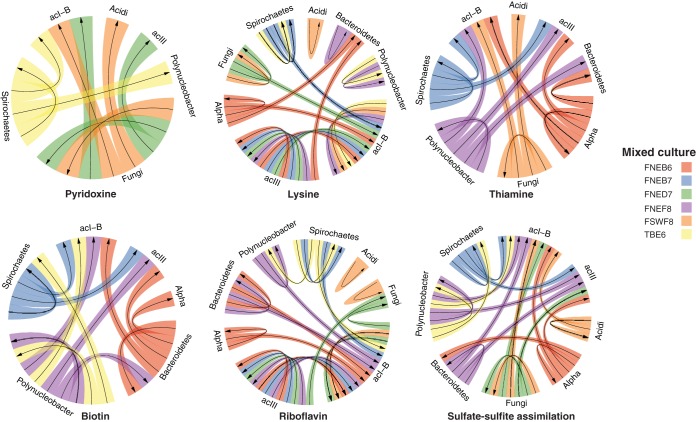
Potential metabolic complementarity among major members of each individual culture. Arrows are based on the presence or absence of biosynthetic pathways. For organisms within the same culture, an arrow points from the organism that has the pathway present to the ones where it is absent. Each circle plot displays one metabolite. Colors indicate each mixed culture. Alpha, alphaproteobacterium; Acidi, *Acidimicrobium*. In the case of pyridoxine, thiamine, and biotin, cultures are left out of the diagram if none of the major members contained the full biosynthetic pathway. Still, some genes of these biosynthetic pathways could be identified in each culture with low coverage.

10.1128/mSphere.00202-18.5 DATA SET S1  Excel spreadsheet (note the tabs in the lower left for Tables 1 and 2). Note that the color coding of the MAGs based on culture origin is consistent throughout and in Fig. 1 in the text. Table 1 shows statistics for all MAGs assembled and binned from the six mixed cultures. Taxonomic classification was determined by inspecting the PhyloPhlAn tree using previously published genomes and SAGs as references. If the MAGs belong to one of the freshwater tribes, they have the name of the tribe. Another taxonomic name is given otherwise. The tribe and taxonomic affiliation were defined using whole-genome information and the public database by using PhyloPhlAn. MAGs were considered major members of a mixed culture when their genome recruited more than 1% of the reads. Completeness was calculated using CheckM. Table 2 shows a symmetrical matrix with ANI values of the main members of the community. Groups that share the same taxonomic group are delineated. Consistent groups are highlighted with gray shading (≥97% ANI). Download DATA SET S1, XLSX file, 0.03 MB. Copyright © 2018 Garcia et al. 2018 Garcia et al. This content is distributed under the terms of the Creative Commons Attribution 4.0 International license.

**TABLE 1:**
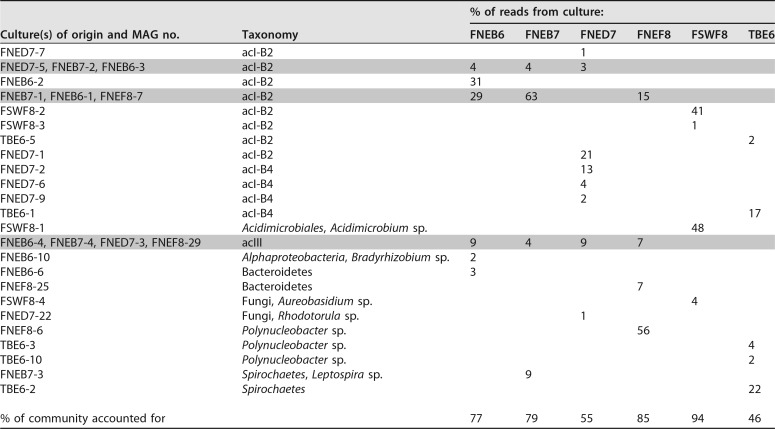
Summary of the main members of the mixed cultures^a^

a The right-most columns represent the 6 cultures, and rows show the different members found. The numbers represent the percentages of reads from the cultures that recruited to the respective MAGs. Shading indicates MAGs that have been clustered together. MAGs were clustered together when they shared more than 97% nucleotide identity. Nucleotide identities are shown in Table 2 in Data Set S1.

The six cultures obtained from three different environments did not resemble each other in composition. As shown in Table 1, different taxa cooccurred with acI actinobacteria in the cultures. For example, *Polynucleobacter* and acIII lineage organisms, which are common freshwater bacteria (10), appeared together with one acI MAG in the culture FNEF8 (13). However, the other cultures from Fuchskuhle’s northeast basin did not contain *Polynucleobacter* and instead contained multiple acI MAGs together with MAGs from the acIII lineage and a few other taxa. To examine whether the different acI MAGs differed in their cooccurrences, MAGs were first clustered into discrete sequence populations using 97% nucleotide identity as a cutoff (9). Overall, only two resulting discrete populations of acI-B2 were found to contain more than one MAG (Table 1). The other acI MAGs represented one sequence of a discrete population each. Also, an acIII discrete population emerged, and it was represented in all cultures from Fuchskuhle’s northeast basin. However, we found no support for a specific linkage between certain discrete populations of acI-B2 and acIII in this basin. Moreover, the more-acidic southwest basin yielded acI MAGs that were distinct from those from the northeast basin, as well as a member of the *Acidimicrobiales*. There is a possibility that free-living microorganisms pick their interaction partners out of a pool of different taxa. However, our observations must be interpreted with caution, as we cannot rule out stochastic effects, such as the dilution bottleneck effect, and phages (16), which can alter the outcome of the cultivation approach toward stochastic community assembly in our mixed cultures.

## AUXOTROPHIES AND METABOLIC DEPENDENCIES BETWEEN DIFFERENT FREE-LIVING MICROORGANISMS

From previously published analyses of complete acI genomes and the acI lineage, auxotrophy for pyridoxine, lysine, thiamine, biotin, riboflavin, and reduced sulfur was observed (4, 11, 17). An analysis of all acI MAGs in this study suggests that most acI members in our mixed cultures also harbor those auxotrophies. At least one of the main members in each community showed the potential to synthesize each of these metabolites, except vitamin B_12_ (Fig. 1). As an example, in culture FNEB6, the alphaproteobacterium is the only member of that culture with the potential to reduce sulfur and so potentially supplies it to the other community members in the form of sulfide or cysteine (Fig. 1). One or two members of each model community had the ability to reduce sulfate, consistent with reports of redox reaction product transfers known as “metabolic handoffs” (18). Since the medium used for growing these cultures was triple-filtered lake water, there is still a possibility that the vitamin B_12_ or traces of other micronutrients support the growth of acI organisms or any other of the members of the model communities.

Since these cultures are a simplification of the complex natural environment, these potential dependencies merely represent a few examples of the many interactions that likely occur in the complex natural environment. Other observed examples of interactions in the environment include a high detoxification ability of a few microorganisms, for example, in the form of catalases and peroxidases (13, 19, 20). In our cultures, the acI members encoded catalase peroxidase, while a few of the other members, such as *Polynucleobacter* and *Acidimicrobiales* organisms, were missing such detoxification genes. Altogether, these observations suggest that interdependencies are the norm rather than the exception. This has implications for constraints on diversity and dynamics arising from the need to rewire interaction networks when one community member supplying a public good declines markedly in abundance (5). Still, it remains unknown whether public goods are actively produced so that the donor cells incur fitness costs or whether the metabolic exchange is merely a result of cohabitation.

## A HIGH DEGREE OF GENOMIC DIVERSITY MIGHT SUPPORT FLEXIBILITY IN INTERACTION PARTNERS

The 33 acI actinobacterial MAGs from our 6 mixed cultures can be assigned to 3 of the 13 recognized tribes, acI-A1, acI-B2, and acI-B4 (Fig. S1). Interestingly, even when our cultures harbored reduced community-level diversity, they all contained more than one acI genotype and even multiple discrete populations as defined by 97% average nucleotide identity (ANI). This reflects a high diversity at the population level of this common and dominant free-living freshwater bacterium (9).

10.1128/mSphere.00202-18.2 FIG S1  Whole-genome amino acid tree of freshwater acI actinobacteria built from a concatenated alignment of 400 single-copy marker genes optimized from among 3,737 genomes using PhyloPhlAn. It includes all publicly available full acI genomes and SAGs and previously published MAGs. In bold are the MAGs from this study. Labels reflect the tribe to which the acI organisms belong as determined by their 16S rRNA and the gene similarity from PhyloPhlAn. Download FIG S1, PDF file, 0.2 MB. Copyright © 2018 Garcia et al. 2018 Garcia et al. This content is distributed under the terms of the Creative Commons Attribution 4.0 International license.

As previous studies with complete genomes have calculated, the acI core genome is about 800 genes (4). Assuming around 1,600 genes per acI organism (21), this means that about half of the whole acI genome belongs to the flexible genome. This is similar to the proportion reported for the family of “*Candidatus* Pelagibacter” (22). Thus, both acI and “*Ca.* Pelagibacter” organisms have larger flexible genomes than photoautotrophic free-living aquatic bacteria like *Prochlorococcus* (8). Just as for the family of “*Ca.* Pelagibacter” (22), the high number of auxiliary genes in populations of streamlined acI genomes is likely to render populations of this free-living bacterial lineage functionally versatile. This versatility combined with auxotrophy creates a fascinating paradox: acI members can do many different things, but they cannot do any of them alone.

## SOME FREE-LIVING MICROORGANISMS FULFILL THEIR METABOLIC NEEDS FROM THOSE THAT THEY HAPPEN TO ENCOUNTER

By diluting and obtaining viable mixed cultures, we first obtained evidence that these plankton community partners represent diverse sets of community members. The variety of different phylotypes growing together with acI hints at a nonspecific metabolic dependence of acI actinobacteria on other abundant freshwater bacteria.

Since six mixed cultures are not sufficient to generalize the character of the observed associations, we correlated the abundance of mixed-culture TBE6 MAGs with newly assembled MAGs from a 9-year shotgun metagenome time series from the mixed-culture’s source environment, Trout Bog Lake (23). If acI actinobacteria do indeed have a nonspecific metabolic dependence on other freshwater bacteria, we expected to see a larger number of positive correlations between the abundance of acI organisms and the abundance of other freshwater bacteria than in the culture. In contrast, a number of positive associations exclusive to taxa recruited in the cultures would indicate highly specific dependencies for acI organisms.

**FIG 2:**
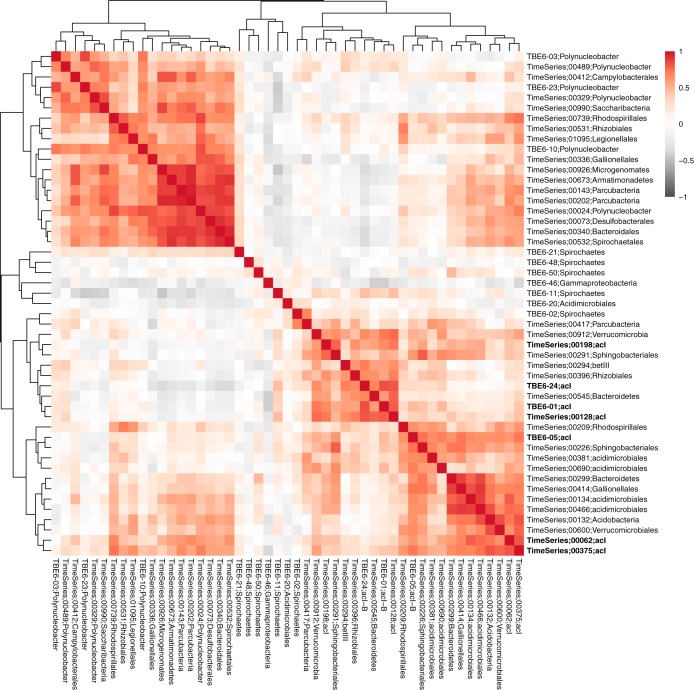
Spearman correlation of normalized relative abundances between MAGs from TBE6 and taxa in the epilimnion of the environment of origin (Trout Bog Lake). Metagenome samples from 45 time points were used. Correlations are sorted using hierarchical clustering. MAG names that start with TBE6 refer to the MAG number from the mixed-culture. MAG names starting with the words “time series” represent bins from a 9-year metagenome time series (23). The acI MAGs are in bold. To view the correlation matrix that was derived from time series metagenomic sequencing of the hypolimnion and from both the epilimnion and the hypolimnion combined, see the supplemental material (Fig. S2 and S3).

In the Trout Bog Lake epilimnion, we recovered 36 MAGs that correlated with the MAGs recovered from the TBE6 culture (Fig. 2). These included four acI MAGs, three *Polynucleobacter* MAGs, and a single *Spirochaetes* MAG that were different (<90% ANI) from those recovered from the mixed culture. The rest of the MAGs had diverse taxonomic affiliations, such as *Saccharibacteria*, *Parcubacteria*, *Verrucomicrobia*, and *Bacteroidetes*, among others. Several positive and negative correlations were observed. In the first quadrant of Fig. 2, positive interactions can be observed, mostly between different taxonomic groups and the *Polynucleobacter* organisms of our TBE6 mixed culture. A second block of positive interactions can be observed between the acI MAGs from TBE6 and diverse taxonomic groups from the lake. Moreover, a third block of positive interactions was detected between one of the acI MAGs from TBE6 and diverse other taxonomic groups, including several distinct acI actinobacteria. Thus, it appears that the positive correlations do not exclusively occur between similar taxonomic groups.

A similar analysis was repeated for the hypolimnion of Trout Bog Lake (see Fig. S2 in the supplemental material) and the combination of both layers (Fig. S3), and similar results were observed. Results from previous cooccurrence studies support the idea that free-living streamlined bacteria have very high connectivities in their environments and are critically dependent on metabolites that might be provided by other planktonic community members (7, 24, 25). Moreover, our analysis shows that in nature, we observe many more interaction partners than we can observe in a single dilution culture. Some future work to confirm the nature of these interactions might include more cultures with the respective supporting time series data and metabolic networks that will confirm the promiscuity of the interactions in free-living microorganisms.

10.1128/mSphere.00202-18.3 FIG S2  Spearman correlation between MAGs from TBE6 and taxa in the hypolimnion of the environment of origin (Trout Bog Lake). Metagenome samples from time points from all available years were used (68 unique dates) to map reads against all 77 available MAGs. Download FIG S2, PDF file, 0.03 MB. Copyright © 2018 Garcia et al. 2018 Garcia et al. This content is distributed under the terms of the Creative Commons Attribution 4.0 International license.

10.1128/mSphere.00202-18.4 FIG S3  Spearman correlation between MAGs from TBE6 and taxa in the epilimnion and hypolimnion of the environment of origin (Trout Bog Lake). Metagenome samples from time points from all available years were used (113 unique dates) to map reads against all 77 available MAGs. Download FIG S3, PDF file, 0.02 MB. Copyright © 2018 Garcia et al. 2018 Garcia et al. This content is distributed under the terms of the Creative Commons Attribution 4.0 International license.

In conclusion, acI actinobacteria appear to depend on numerous other abundant microorganisms for metabolic handouts (i.e., some vitamins, amino acids, and reduced sulfur). This kind of dependence seems to be non-taxon specific or promiscuous since highly specific exclusive cooccurrences could not be observed in the mixed cultures or in the time series metagenomes. This, we hypothesize, might also be the case with other free-living streamlined organisms. Paradoxically, with a small genome size, the large proportion of accessory genes renders them functionally versatile at the population level. Our results suggest that this metabolic versatility facilitates interactions with a variable set of community members. In natural systems with a high temporal and spatial variability in environmental drivers, this might be one of the keys to the competitiveness of streamlined “free-living” microorganisms in highly dynamic microbial communities.

### Accession number(s).

The raw shotgun metagenome reads are publicly available in the JGI portal, and the assembly is available in the IMG database under the submission numbers 26656, 26658, 26650, 29729, 29808, and 50227. The bacterial metagenome-assembled genomes (MAGs) are also available through IMG. The MAGs assembled from Trout Bog Lake (GGBR00000000) and fungal MAGs have been deposited in DDBJ/ENA/GenBank. For taxon operational identifiers or accession numbers of MAGs from mixed cultures, see the supplemental material.

10.1128/mSphere.00202-18.1 TEXT S1  Supplemental materials and methods. Download TEXT S1, DOCX file, 0.2 MB. Copyright © 2018 Garcia et al. 2018 Garcia et al. This content is distributed under the terms of the Creative Commons Attribution 4.0 International license.
