# Supplementary figures and images for: Model Communities Hint at Promiscuous Metabolic Linkages between Ubiquitous Free-Living Freshwater Bacteria
Source: mSphere. 2018 May 30;3(3):e00202-18. doi: 10.1128/mSphere.00202-18 (PMC5976882; doi:10.1128/mSphere.00202-18)

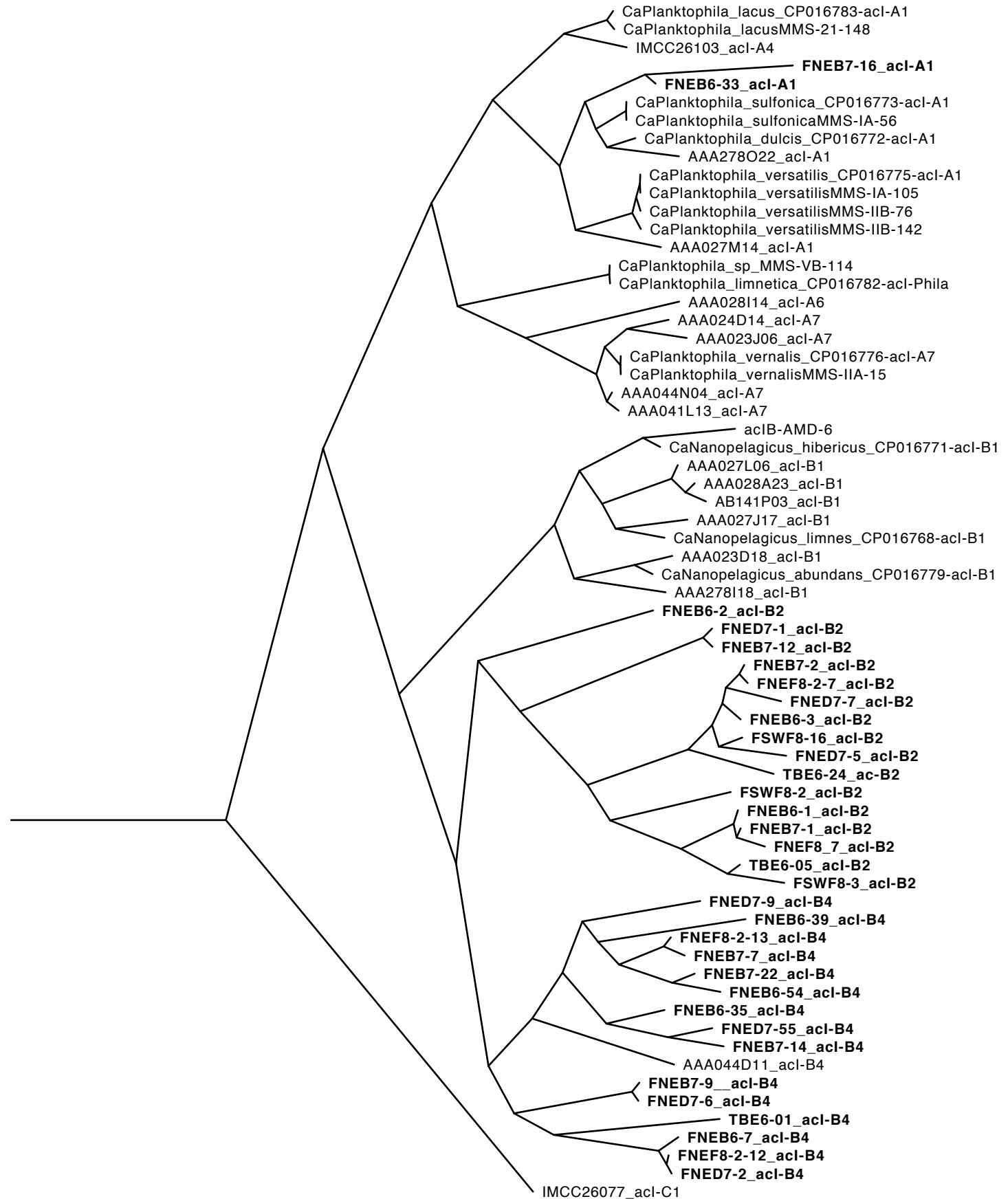

0.09

Supplement: FIG S1 [file sph003182557sf1.pdf]

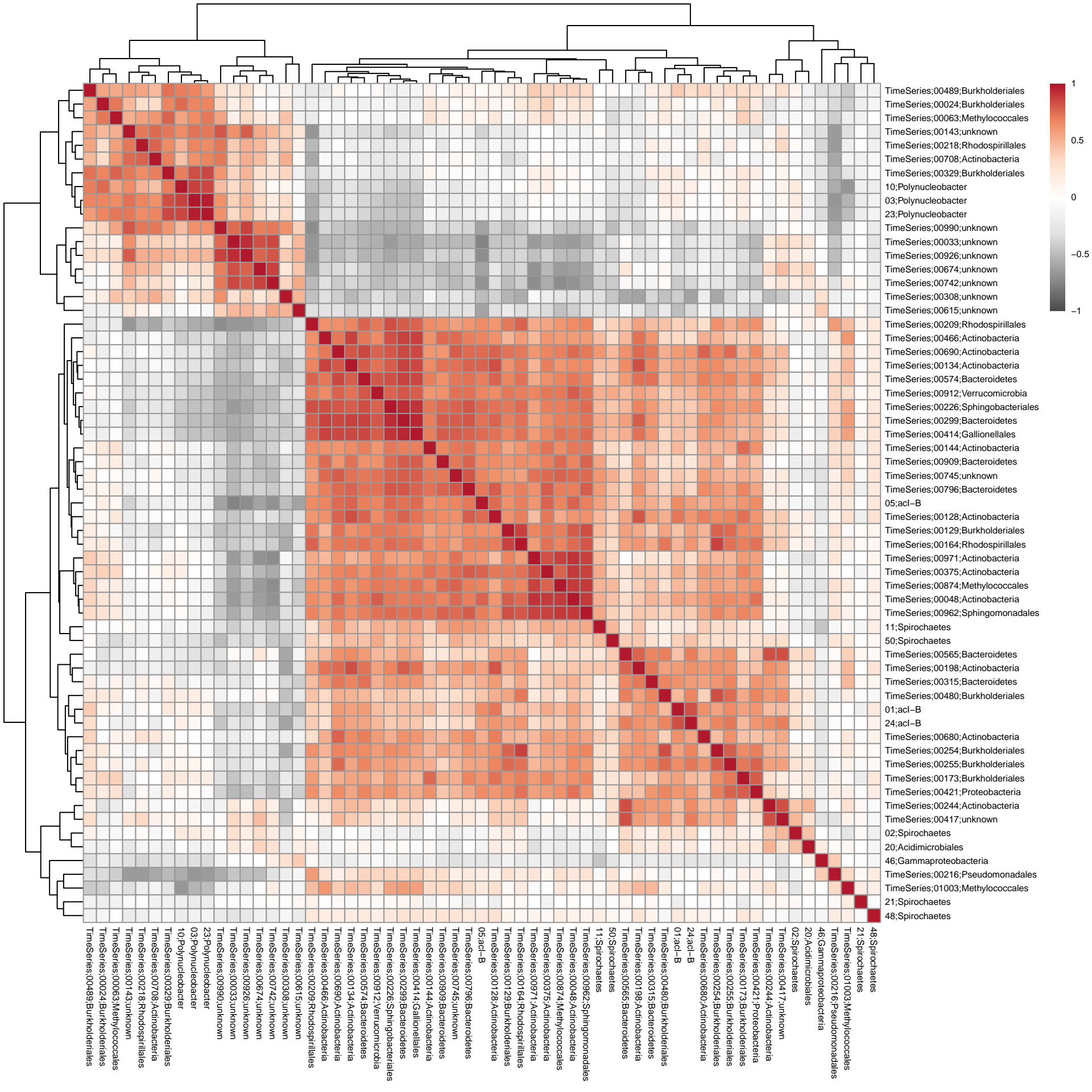

Supplement: FIG S2 [file sph003182557sf2.pdf]

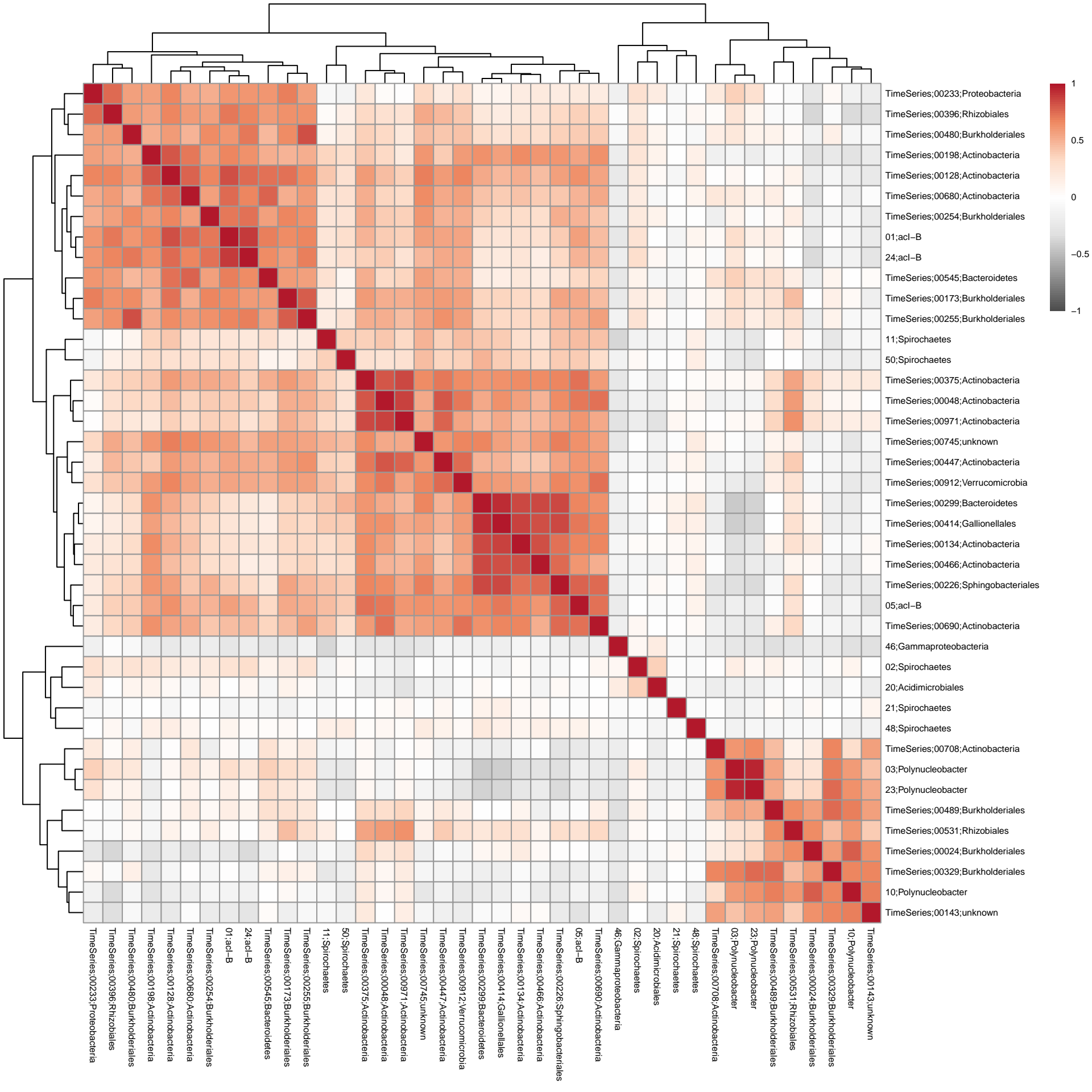

Supplement: FIG S3 [file sph003182557sf3.pdf]
