# Supplementary material for: Model Communities Hint at Promiscuous Metabolic Linkages between Ubiquitous Free-Living Freshwater Bacteria
Source: mSphere. 2018 May 30;3(3):e00202-18. doi: 10.1128/mSphere.00202-18 (PMC5976882; doi:10.1128/mSphere.00202-18)
Supplement: TEXT S1 [file sph003182557s1.docx]

**Materials and Methods**

*Mixed culture and DNA extraction*

Mixed cultures that included the abundant and ubiquitous freshwater *Actinobacteria* were established in 2012 and 2013. Samples were collected from 2 different lakes: Lake Grosse Fuchskuhle (North East basin and South West basin), and from Trout Bog Lake (Supplementary Tab Lakes-metadata). In total, four plates were set up, two for Northeast basin (Lake Grosse Fuchskuhle) and one for each of the other lakes (Southwest basin Lake Grosse and Trout Bog Lake). Cultivation method, media preparation, acI qPCR assays, general observations and DNA extractions were as previously described for mixed culture FNE-F8 (1). In brief, lake water was triple filtered through 0.2µm filters. Cells were diluted in this water and grown for up to 30 days. They were then scaled up in more triple filtered water and grown for seven days, which is when they reached maximum density. Selected mixed cultures (based on cell density) were scaled up to a four-liter culture and DNA was extracted. DNA was sent to JGI for shotgun metagenomic sequencing.

*Library preparation and sequencing*

JGI performed the library preparation and the sequencing under Community Sequencing Project 1289. First, 100 ng of genomic DNA was sheared to 270 bp using a focused-ultrasonicator (Covaris). The sheared DNA fragments were size selected using SPRI beads (Beckman Coulter). The selected fragments were then end-repaired, A-tailed, and ligated of Illumina compatible adapters (IDT, Inc.) using KAPA-Illumina library creation kit (KAPA biosystems). The prepared sample library was quantified using KAPA Biosystem’s next-generation sequencing library qPCR kit and run on a Roche LightCycler 480 real-time PCR instrument. The quantified library was then prepared for sequencing on the Illumina HiSeq sequencing platform utilizing a TruSeq paired-end cluster kit, v3, and Illumina’s cBot instrument to generate a clustered flowcell for sequencing. Sequencing of the flowcell was performed on the Illumina HiSeq2000 sequencer using Illumina TruSeq SBS sequencing kits, v3, following a 2x150 indexed high-output run recipe.

*Genome assembly, annotations and metabolic features analysis*

After reads had been filtered based on their quality scores using sickle (version 1.210) (2), the reads were digitally normalized using khmer 1.4 (3) and assembled with megahit (4). Coverage was computed by mapping back the reads competitively to the assembly using bbmap 35.40 using defult settings (sourceforge.net/projects/bbmap/) using default parameters. Mapped reads were indexed and sorted using SAMtools 1.3 (5) while we removed duplicates using picard-tools (version 1.101). Bedtools (version 2.18.2) (6) was used for computing coverage. For the binning metaBAT (7) was used. Tribe/taxonomical affiliation were defined using whole genome information and the public database using PhyloPhlAn (8) and using previously published genomes and SAGs (9) as references. If the MAGs belong to one of the freshwater tribes (10) they have the name of the tribe, otherwise the taxonomic name after NCBI is given.

For bacterial genomes, gene prediction analysis was performed within the Integrated Microbial Genomes (IMG) (11, 12) platform developed by the Joint Genome Institute, Walnut Creek, CA, USA (<http://img.jgi.doe.gov>). Specific KEGG biosynthetic maps were inspected for completeness (<http://www.genome.jp/kegg/mapper.html>) counting number of missing enzymes from the most complete pathway. MAG completeness was calculated using CheckM (13). ANI was calculated as previously described (14, 15). Based on average MAG completeness, completeness of a metabolic pathway was considered if 80% of the pathway was present.

For fungal genomes, ab initio gene prediction was performed using augustus (v 2.5.5) with Aspergillus nidulans (FSWF8-4) or Cryptococcus neoformans (FNED7-22) models. Gene model ORFs were then annotated using matches to Pfam 30.0 (June 2016) by hmmscan (HMMER 3.1b2). Functional annotation was done using predicted aminoacids in the KAAS platform (16).

*Metagenome recruitment to MAGs*

A total of 113 metagenome samples from Trout Bog lake (17) were used in this study. Genomes were assembled and binned from these lake metagenomes as described for the cultures. bbmap was used to compute the coverage of all the MAGs (from the time-series as well as the cultures) using default settings, for all the time-points. All coverages have been normalized to metagenomic sequencing depth. Mean coverage vectors of all MAGs were then correlated using Spearman correlation. For the heatmaps, MAGs from Trout Bog Lake were only included if they correlated with at least 0.6 to at least one MAG from our mixed culture TBE6 (positive or negative correlation). MAGs assembled from Trout Bog Lake have not been further analyzed here, though they are the subject of other detailed studies completed (18) and ongoing.

**References**

1. Garcia SL, McMahon KD, Grossart HP, Warnecke F. 2014. Successful enrichment of the ubiquitous freshwater acI Actinobacteria. Environ Microbiol Rep 6:21-7.

2. Joshi NA, Fass JN. Sickle: A sliding-window, adaptive, quality-based trimming tool for FastQ files [Software]. Available at <https://githubcom/najoshi/sickle>.

3. Crusoe MR, Alameldin HF, Awad S, Boucher E, Caldwell A, Cartwright R, Charbonneau A, Constantinides B, Edvenson G, Fay S, Fenton J, Fenzl T, Fish J, Garcia-Gutierrez L, Garland P, Gluck J, Gonzalez I, Guermond S, Guo J, Gupta A, Herr JR, Howe A, Hyer A, Harpfer A, Irber L, Kidd R, Lin D, Lippi J, Mansour T, McA'Nulty P, McDonald E, Mizzi J, Murray KD, Nahum JR, Nanlohy K, Nederbragt AJ, Ortiz-Zuazaga H, Ory J, Pell J, Pepe-Ranney C, Russ ZN, Schwarz E, Scott C, Seaman J, Sievert S, Simpson J, Skennerton CT, Spencer J, Srinivasan R, Standage D, et al. 2015. The khmer software package: enabling efficient nucleotide sequence analysis. F1000Res 4:900.

4. Li D, Liu CM, Luo R, Sadakane K, Lam TW. 2015. MEGAHIT: an ultra-fast single-node solution for large and complex metagenomics assembly via succinct de Bruijn graph. Bioinformatics 31:1674-6.

5. Li H, Handsaker B, Wysoker A, Fennell T, Ruan J, Homer N, Marth G, Abecasis G, Durbin R, Genome Project Data Processing S. 2009. The Sequence Alignment/Map format and SAMtools. Bioinformatics 25:2078-9.

6. Quinlan AR, Hall IM. 2010. BEDTools: a flexible suite of utilities for comparing genomic features. Bioinformatics 26:841-842.

7. Kang DD, Froula J, Egan R, Wang Z. 2015. MetaBAT, an efficient tool for accurately reconstructing single genomes from complex microbial communities. PeerJ 3:e1165.

8. Segata N, Bornigen D, Morgan XC, Huttenhower C. 2013. PhyloPhlAn is a new method for improved phylogenetic and taxonomic placement of microbes. Nat Commun 4:2304.

9. Garcia SL, Stevens SLR, Crary B, Martinez-Garcia M, Stepanauskas R, Woyke T, Tringe SG, Andersson SGE, Bertilsson S, Malmstrom RR, McMahon KD. 2018. Contrasting patterns of genome-level diversity across distinct co-occurring bacterial populations. ISME J 12:742-755.

10. Newton RJ, Jones SE, Eiler A, McMahon KD, Bertilsson S. 2011. A Guide to the Natural History of Freshwater Lake Bacteria. Microbiol Mol Biol Rev 75:14-49.

11. Markowitz VM, Chen IM, Palaniappan K, Chu K, Szeto E, Pillay M, Ratner A, Huang J, Woyke T, Huntemann M, Anderson I, Billis K, Varghese N, Mavromatis K, Pati A, Ivanova NN, Kyrpides NC. 2014. IMG 4 version of the integrated microbial genomes comparative analysis system. Nucleic Acids Res 42:D560-7.

12. Markowitz VM, Chen IM, Chu K, Szeto E, Palaniappan K, Pillay M, Ratner A, Huang J, Pagani I, Tringe S, Huntemann M, Billis K, Varghese N, Tennessen K, Mavromatis K, Pati A, Ivanova NN, Kyrpides NC. 2014. IMG/M 4 version of the integrated metagenome comparative analysis system. Nucleic Acids Res 42:D568-73.

13. Parks DH, Imelfort M, Skennerton CT, Hugenholtz P, Tyson GW. 2015. CheckM: assessing the quality of microbial genomes recovered from isolates, single cells, and metagenomes. Genome Res doi:10.1101/gr.186072.114.

14. Goris J, Konstantinidis KT, Klappenbach JA, Coenye T, Vandamme P, Tiedje JM. 2007. DNA-DNA hybridization values and their relationship to whole-genome sequence similarities. International Journal of Systematic and Evolutionary Microbiology 57:81-91.

15. Rodriguez-R LM, Konstantinidis KT. 2014. Bypassing cultivation to identify bacterial species. Microbe 9.

16. Moriya Y, Itoh M, Okuda S, Yoshizawa AC, Kanehisa M. 2007. KAAS: an automatic genome annotation and pathway reconstruction server. Nucleic Acids Research 35:W182-W185.

17. Bendall ML, Stevens SL, Chan LK, Malfatti S, Schwientek P, Tremblay J, Schackwitz W, Martin J, Pati A, Bushnell B, Froula J, Kang D, Tringe SG, Bertilsson S, Moran MA, Shade A, Newton RJ, McMahon KD, Malmstrom RR. 2016. Genome-wide selective sweeps and gene-specific sweeps in natural bacterial populations. ISME J doi:10.1038/ismej.2015.241.

18. Hamilton JJ, Garcia SL, Brown BS, Oyserman BO, Moya-Flores F, Bertilsson S, Malmstrom RR, Forest KT, McMahon KD. 2017. Metabolic Network Analysis and Metatranscriptomics Reveal Auxotrophies and Nutrient Sources of the Cosmopolitan Freshwater Microbial Lineage acI. mSystems 2.
